# Determination the Usefulness of *AhHMA4p1::AhHMA4* Expression in Biofortification Strategies

**DOI:** 10.1007/s11270-016-2877-0

**Published:** 2016-05-23

**Authors:** Aleksandra Weremczuk, Anna Barabasz, Anna Ruszczyńska, Ewa Bulska, Danuta Maria Antosiewicz

**Affiliations:** Faculty of Biology, Institute of Experimental Plant Biology and Biotechnology, University of Warsaw, Miecznikowa str 1, 02-096 Warszawa, Poland; Faculty of Chemistry, University of Warsaw, Pasteura str. 1, 02-093 Warszawa, Poland

**Keywords:** Biofortification, Cadmium, HMA4, Tomato, Transformation, Zinc

## Abstract

*AhHMA4* from *Arabidopsis thaliana* encodes Zn/Cd export protein that controls Zn/Cd translocation to shoots. The focus of this manuscript is the evaluation of *AhHMA4* expression in tomato for mineral biofortification (more Zn and less Cd in shoots and fruits). Hydroponic and soil-based experiments were performed. Transgenic and wild-type plants were grown on two dilution levels of Knop’s medium (1/10, 1/2) with or without Cd, to determine if mineral composition affects the pattern of root/shoot partitioning of both metals due to *AhHMA4* expression. Facilitation of Zn translocation to shoots of 19-day-old transgenic tomato was noted only when plants were grown in the more diluted medium. Moreover, the expression pattern of Zn-Cd-Fe cross-homeostasis genes (*LeIRT1*, *LeChln*, *LeNRAMP1*) was changed in transgenics in a medium composition-dependent fashion. In plants grown in soil (with/without Cd) up to maturity, expression of *AhHMA4* resulted in more efficient translocation of Zn to shoots and restriction of Cd. These results indicate the usefulness of *AhHMA4* expression to improve the growth of tomato on low-Zn soil, also contaminated with Cd.

**Electronic supplementary material:**

The online version of this article (doi:10.1007/s11270-016-2877-0) contains supplementary material, which is available to authorized users.

## Introduction

Zinc (Zn) is an essential microelement for living organisms. Its deficiency in plants retards growth and reduces crop yields. Consequently, plant-derived foods are low in Zn, thus contributing to Zn malnutrition in humans, and Zn deficiency is a major risk factor in human health (Welch & Graham 2004). On the other hand, cadmium (Cd), a non-essential element for plants, when present in the soil/medium can become highly toxic. Cd is taken up by roots through transporters for essential elements, primarily Zn, Fe, and Ca (Clemens 2006). Among others, its presence deregulates the homeostasis of essential metals, disturbs their uptake, or displaces them from proteins (Verbruggen et al. 2009). Cd accumulates in vegetative and reproductive organs, including edible plant parts, thus entering the food chain. The major source of Cd for humans is plant-based food. Even low doses of Cd present in food, when taken on a regular basis, accumulate in the body over time, posing a threat for the development of Cd-related diseases (Palmgren et al. 2008).

Mineral malnutrition can be addressed through dietary diversification, mineral supplementation, or biofortification. Biofortification in Zn aims at increasing its concentration in plant-derived food through improving the efficiency of uptake from soil and translocation to edible parts of crop plants. On the other hand, reducing the content of toxic metals like Cd is needed. In engineering improved mineral quality of edible aerial plant parts, the critical factor is the efficiency of metal root-to-shoot translocation (enhancement of micronutrients, decrease of toxic metals). A transgenic approach involves, among others, ectopic expression of transporters responsible for loading metals into xylem vessels in roots, which determines the efficiency of the translocation process (Palmgren et al. 2008).

Root-to-shoot translocation of Zn (and Cd) not only in *Arabidopsis thaliana*, *Arabidopsis halleri*, but also in *Thlaspi caerulescens* and tobacco, depends on expression of *HMA4* (a P_1B_-ATPase) encoding a plasma membrane-localized efflux protein that participates in Zn/Cd xylem loading (Bernard et al. 2004; Hussain et al. 2004; Papoyan & Kochian 2004; Verret et al. 2004; Hanikenne et al. 2008; Wong & Cobbett 2009; Hermand et al. 2014). In an *AtHMA4* knockout mutant of *A. thaliana*, translocation of both metals was impaired, leading to their decreased accumulation in leaves (Hussain et al. 2004; Verret et al. 2004). In the Zn hyperaccumulator, the unusual ability of *A. halleri* (accumulates Zn in leaves up to 3 % of their dry weight) to transfer high amounts of Zn to shoots is conferred by tenfold higher expression of *AhHMA4* than in the non-accumulating *A. thaliana* (Talke et al. 2006; Hanikenne et al., 2008). For comparison, downregulation of *AhHMA4* expression by RNA interference dramatically decreased Zn and Cd concentrations in the shoots and increased them in the roots (Hanikenne et al. 2008).

Considering the role that HMA4 plays in the control of Zn and Cd translocation to shoots, *AtHMA4* under the 35SCaMV promoter was used to transform tobacco to engineer high metal contents in the shoots for phytoremediation purposes (Siemianowski et al. 2011). Expression of *AtHMA4* resulted in an increased Zn level in the shoots (although this effect was not seen at all applied Zn exposures) and, unexpectedly, in a decreased Cd level. Similarly, expression of *AhHMA4p1::AhHMA4* from *A. halleri* in tomato facilitated root-to-shoot Zn translocation and induced Zn uptake in a Zn-supply dependent manner (Barabasz et al. 2012).

Further studies showed that ectopic expression of *AtHMA4* in tobacco did not mimic the physiological role it plays in *A. thaliana*. Instead, modifications of endogenous tobacco metal homeostasis mechanisms took place as a response to disturbed homeostasis of Zn, Fe, and also other metals due to the export activity of AtHMA4 in all cells of the transgenic plant. It was concluded that these alterations were a key factor in generating the metal-related phenotype of *HMA4*-expressing plants (changes in the uptake/distribution of a metal/s) (Barabasz et al., 2012; Siemianowski et al., 2013; Antosiewicz et al., 2014). It is known that proteins involved in the regulation of metal metabolism (transport and chelating processes) are not highly specific for one substrate. Since they bind several metals with different affinity, the same protein is involved in the regulation of homeostasis of several metals. The contribution of the same protein to the regulation of the metabolism of different metals is referred to as cross-homeostasis. As a result, excess or deficiency of a particular metal can influence distribution or translocation of other metals and affect overall plant growth and development. These processes have been emphasized in reports that changes in micronutrient homeostasis are involved in the toxicity of Zn or Ni excess (Ghasemi et al. 2009; Barabasz et al. 2012). Their involvement in the alleviation of toxicity of Cd, Pb, or other metals by application of excess Ca, Mg, or Zn has also been reported (Kinraide et al. 2004; Antosiewicz & Hennig 2004; Antosiewicz 2005; Wojas et al. 2007). In our studies, induction of the Zn–Fe cross-homeostasis network to reconstitute the ion balance was reported in tomato expressing *AhHMA4p1::AhHMA4*. In these plants, transgene expression led to enhanced Fe deficiency status (relative to wild-type plants) which was triggered by exposure to excess Zn (Barabasz et al., 2012; Antosiewicz et al., 2014).

In this study, the usefulness of *AhHMA4p1::AhHMA4* from *A. halleri* in transformation for the purpose of biofortification was examined (to increase the Zn and decrease the Cd contents in aerial plant parts, including fruits and seeds). Tomato expressing *AhHMA4p1::AhHMA4* (generated and initially characterized by Barabasz et al. 2012) was used as the experimental material. Considering the detected Zn supply-dependent modification pattern of Zn root/shoot distribution in *AhHMA4*-expressing plants, the aim was also to determine the possible dependence of the modification of a metal’s root/shoot distribution in transgenic plants on the overall mineral composition of the medium. Plants were grown in the presence or absence of Cd in hydroponic media of different mineral compositions until the stage of young seedlings and on soil until maturation and fruit ripening.

## Materials and methods

### Plant material and general growth conditions

The experiments were conducted on the following lines of tomato (*Lycopersicon esculentum* L. cv. Money Maker.): (i) wild type and (ii) two independent transgenic homozygous lines (no. 6 and no. 8) with the expression of the *AhHMA4p1::AhHMA4* at the similar level (Barabasz et al. 2012). The plants were cultivated in a growth chamber (16 h light, temperature 23/16 °C day/night, 40−50 % humidity) at a quantum flux density (PAR) of 250 μmol m^−2^ s^−1^ using fluorescent Flora tubes.

Seeds were surface sterilized in 70 % ethanol for 30 s, then washed with sterile water. Germination and the first phase of growth were conducted on 1/4 Knop’s medium supplemented with 2 % sucrose and solidified with 0.8 % agar, in glass jars covered with ‘closure-caps’ to keep the conditions sterile and allow ventilation (Barabasz et al. 2012). For the first 4 days, the jars were kept in the dark (until germination), then for 1 week under light (until the first leaves emerged). The obtained 1-week-old seedlings were used for further growth either under hydroponic conditions or in soil (as described below).

### Hydroponic experiments

One-week-old seedlings were transferred from glass jars to 2.4-L containers containing aerated hydroponic solutions to expose them to the following treatments: (i) pre-culture in 1/4 Knop’s medium for 2 days (to adjust to hydroponic conditions), (ii) growth on 1/10 or 1/2 Knop’s medium for 6 days (nutrient solution refreshed every 3 days), followed by 4-day growth under the same conditions (1/10 or 1/2 Knop’s medium) with or without the presence of 0.25 μM Cd added as CdCl_2_. The composition of the full Knop’s medium is as follows: 3 mM Ca(NO_3_)_2_, 1.5 mM KNO_3_, 1.2 mM MgSO_4_, 1.0 mM KH_2_PO_4_, microelements: 40 μM NaFeEDTA, 25 μM H_3_BO_3_, 2.0 μM MnCl_2_, 2.0 μM ZnSO_4_, 0.1 μM CuSO_4_, 0.5 μM Na_2_MoO_4_, 5.0 μM KJ, and 0.1 μM CoCl_2_ (Barabasz et al. 2010). Here, depending on the experiment, the concentration of all elements was reduced up to 1/2 or 1/10 of the full Knop’s medium. Six plants were grown in each pot; three containers were used for each experimental regimen.

At the end of each experiment, the plants’ appearance was assessed visually. Roots and shoots were separated, leaves were cut off, and all plant parts were dried in an oven at 56 °C for 3 days. The dry biomass was determined. Collected samples were used for evaluation of Cd, Zn, and Fe concentrations in roots and leaves.

Roots and leaves (2nd and 3rd counting upward) were also collected for determination of the expression level of selected metal-homeostasis genes (see “Statistical analysis”). For that purpose, roots and leaves were immediately frozen in liquid nitrogen and stored at −80 °C until analysis.

### Soil experiments

Plants were grown in control soil (commercial soil, Krönen) and in soil to which Cd was added (10 mg Cd/kg dry matter of soil) as a CdCl_2_ solution. Afterwards, the soil was kept for 2 weeks before planting with daily mixing and dampening with equal amounts of distilled water according to Wojas et al. (2007).

One-week-old seedlings were transferred from glass jars to liquid 1/4 Knop’s medium and grown for 2 weeks. Three-week-old plants were transferred for further growth to pots containing soil with or without the addition of Cd, one plant/pot containing 1.35 kg of soil. Plants were grown on soil for 101 days. Five plants were used for each experimental regimen. The plants were watered regularly with distilled water. The side shoots were removed on a regular basis. Only two inflorescences were left on the main shoot. The growth and development of plants were monitored throughout the experiments, and their appearance was assessed visually. At the end of the experiments, the roots and shoots were separated. Leaves were cut off the stem and collected in two groups: (i) lower leaves (leaves 1 to 7 counting upward) and (ii) upper leaves (remaining leaves above the “lower leaves”). Roots were thoroughly washed under tap water, then in distilled water. Fruits were collected upon maturity throughout the whole growth period. Seeds were removed from ripe fruits, washed, gently dried with filter paper, and counted. Collected plant parts were dried in an oven at 56 °C, and their dry biomass was determined. Dried plant material was used for the determination assay of Cd, Zn, and Fe concentrations.

### Determination of Cd, Zn, and Fe concentrations in dried plant material and in soil

Dried plant parts (roots, leaves, fruits, seeds; see sections “Hydroponic experiments” and “Soil experiments”) were wet-ashed in a closed system microwave mineralizer (Milestone Ethos 900, Milestone, Bergamo, Italy), as described by Wojas et al. (2009). The Cd, Zn, and Fe concentrations in plant samples were determined by flame atomic absorption spectrophotometry (TJA Solution Solar M, Thermo Electron Manufactured Ltd., Cambridge, Great Britain). The certified reference material for the analysis of the plant parts (Virginia tobacco leaves; PVTL; IChTJ, Poland) was included in each analysis run. In the soil, the pseudo-total concentration of metals was determined by inductively coupled plasma mass spectrometry (ICP-MS, Model Elan 9000, Perkin-Elmer Sciex, Canada) as described by Antosiewicz et al. (2008).

### Real-time qPCR analysis of gene expression

The levels of gene expression were evaluated in the roots and leaves collected from hydroponically grown plants (“Hydroponic experiments”). The RNA isolation and quantitative real-time expression analysis were performed as described by Siemianowski et al. (2014). The sequences of primers are given in Table 1. For each sample, reactions were set up in triplicate. The tomato *LeCyP* (*cyclophiline*) gene was used as the reference gene.

**Table 1:** Primer sequences used for expression analysis

Gene name	Accession number	Primer sequences	Product size (nt)
*LeIRT1*	AF136579	F: TCCTACAGGCGGAGTATAAG	138
		R: CAGTTATTAACGCCCGTGGA	
*LeChln*	AJ242045	F: GGTGCCTATGTGTTACCTAG	106
		R: CTCACTCACTCCTCCAGAG	
*LeNramp1*	AY196091	F: CACTCTGCCCTTGTACTATC	142
		R: GTACCAGACACAGACACAAC	
*LeZIP4*	XM_004245052.2	F: CCATTGGTTGGCAAGAAGC	158
		R: GGAGATTTCGAAGACAAGG	
*AhHMA4*	DQ221101	F: CTGTGACGCTTCTCTTTTTCC	145
		R: CTGATCTGGTGCTATCTGAC	
*LeCyph*	NM_001247559	F: CTCTTCGCCGATACCACTCC	120
		R: TCACACGGTGGAAGGTTGAG	

### Statistical analysis

Statistical significance was calculated at the 0.05 probability level using Student’s *t* test. Analysis was performed with Excel 2007 for Windows.

## Results

### Dependence of the metal-related phenotype of transgenic plants on medium composition

In performed experiments, highly diluted medium (1/10 Knop’s medium) versus rich medium (1/2 Knop’s) were used to simulate soil poor and rich in nutrients and to evaluate a possible contribution of the expression of *AhHMA4p1::AhHMA4* to a better performance of transgenic plants under low mineral medium composition with more focus on root-borne processes. In soil experiments, it is not possible to examine in roots precisely metal accumulation and expression of chosen genes (the majority of fine roots are lost when soil is removed from the root system). Therefore, the experimental setup used in this study involved simulation of low and rich medium with the use of hydroponic experiments. In plants grown hydroponically, metals are available for uptake (as from soil solution), and applied concentrations are comparable to those identified in the soil solution of agricultural soils. For example, in soil solutions extracted from variety of soils, Zn concentrations varied between 0.14 and 33 μM (Del Castillo et al., 1993), whereas in our study 0.2–1 μM.

#### Growth

Previous study showed that the pattern of Zn root/shoot distribution modified due to expression of *AhHMA4* varied over a range of Zn supply (Barabasz et al. 2012). To further characterize the phenomenon of medium composition-dependent modification of Zn root/shoot distribution in *AhHMA4*-expressing tomato, metal accumulation was determined in transgenic and wild-type plants cultivated for 10 days on 1/10 and 1/2 strength Knop’s medium with and without 0.25 μM Cd.

In comparison with the wild type, transgenic tomato expressing *AhHMA4p1::AhHMA4* did not show any distinguishable differences in visual appearance, both in the presence and absence of Cd (data not shown). In all experimental trials, the plants had green healthy looking leaves, and exposure to 0.25 μM Cd did not induce any visual symptoms of toxicity (data not shown). Roots and leaves biomass production remained at a similar level in transgenic and wild-type plants. A difference was detected, however, for leaves between all lines grown on 1/10 and on 1/2 Knop’s medium. Their biomass was lower for those grown on more diluted medium (Online Resource 1).

#### Zn, Cd, and Fe accumulation

**Fig. 1:**
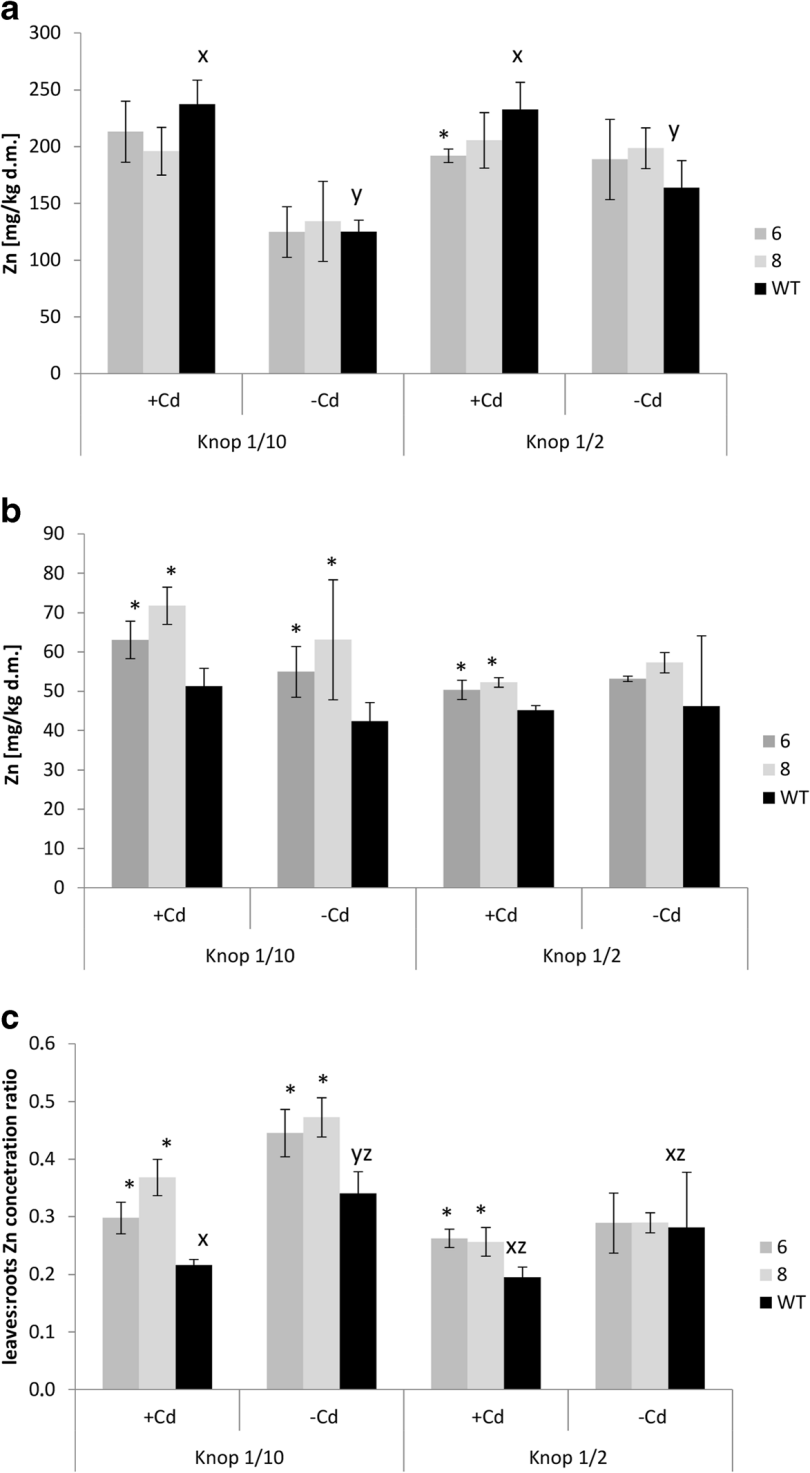
Zn concentration in tomato plants expressing *AhHMA4* (*lines 6 and 8*) and wild type (*WT*). Zn concentration in the roots (**a**), leaves (**b**), and leaves/roots Zn concentration ratio (**c**) of 19-day-old plants grown hydroponically in 1/2 and 1/10 Knop’s medium without Cd or exposed to 0.25 μM Cd for 4 days. Values correspond to arithmetic means ± SD (*n* = 6); values for transgenic plants significantly different from WT at each experimental variant are highlighted by an *asterisk* (*P* ≤ 0.05). *Different letters* represent significantly different values at *P* ≤ 0.05 for wild-type plants grown upon different medium composition (evaluated by Student’s *t* test)

Expression of *AhHMA4* led to increased Zn concentrations in the leaves of plants grown on the more dilute, 1/10 Knop’s medium regardless of the presence or absence of Cd (Fig. 1), which resulted in significantly higher leaves:roots Zn ratio, indicating facilitation of Zn transfer from the roots to shoots. This effect, however, was highly reduced in plants grown on the higher, 1/2 Knop’s medium (Fig. 1b). Under these conditions, the efficiency of Zn transfer to aerial parts of plants grown on medium without Cd did not differ from the wild-type, but in the presence of Cd a slight increase (relative to the wild-type) was still visible (Fig. 1c).

**Fig. 2:**
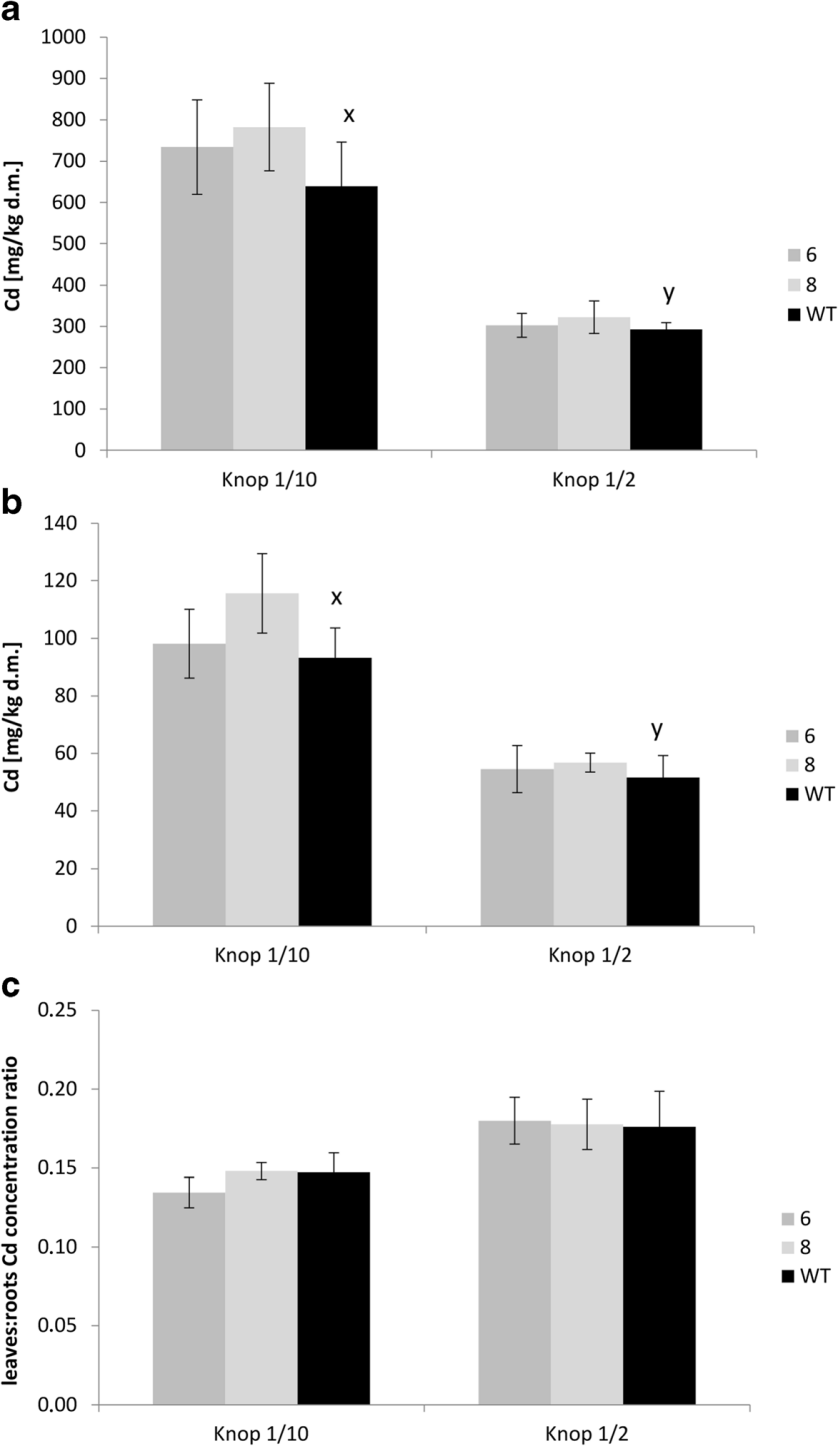
Cd concentration in tomato plants expressing *AhHMA4* (*lines 6 and 8*) and wild type (*WT*). Zn concentration in the roots (**a**), leaves (**b**), and leaves/roots Cd concentration ratio (**c**) of 19-day-old plants grown hydroponically in 1/2 and 1/10 Knop’s medium without Cd or exposed to 0.25 μM Cd for 4 days. Values correspond to arithmetic means ± SD (*n* = 6). *Different letters* represent significantly different values at *P* ≤ 0.05 for wild-type plants grown upon different medium composition (evaluated by Student’s *t* test)

Cd accumulation and the root/shoot distribution pattern in wild-type plants grown on both tested medium compositions were not affected by expression of *AhHMA4* (Fig. 2). The concentrations as well as leaves/roots Cd ratio were comparable for all tested lines grown on medium containing two different levels of nutrients. However, the Cd concentration for both transgenic and wild-type plants grown on 1/10 Knop’s medium was significantly higher compared with 1/2 Knop’s medium (Fig. 2a, b), and the Cd leaves/roots ratio for all lines was slightly lower (Fig. 2c). These findings indicate less efficient uptake of Cd in the presence of higher concentrations of trace- and macroelements (in 1/2 Knop’s), but moderately more efficient translocation to shoots.

**Fig. 3:**
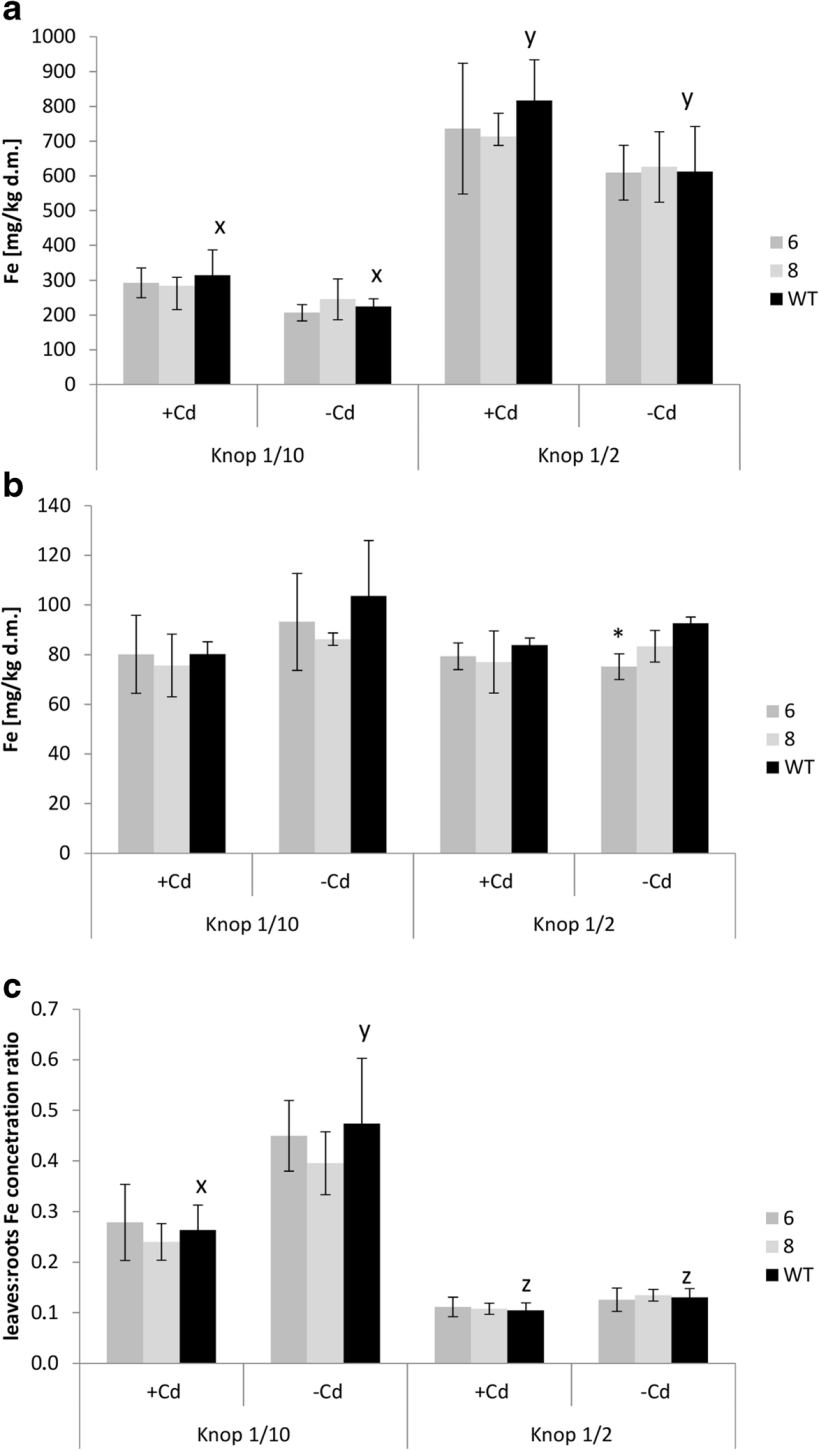
Fe concentration in tomato plants expressing *AhHMA4* (*lines 6 and 8*) and wild type (*WT*). Fe concentration in the roots (**a**), leaves (**b**), and leaves/roots Fe concentration ratio (**c**) of 19-day-old plants grown hydroponically in 1/2 and 1/10 Knop’s medium without Cd or exposed to 0.25 μM Cd for 4 days. Values correspond to arithmetic means ± SD (*n* = 6); values for transgenic plants significantly different from WT is highlighted by an *asterisk* (*P* ≤ 0.05). *Different letters* represent significantly different values at *P* ≤ 0.05 for wild-type plants grown upon different medium composition (evaluated by Student’s *t* test)

A previous study showed that expression of *AhHMA4* in tomato interfered in the Fe metabolism of plants exposed to a range of Zn levels (Barabasz et al. 2012); therefore, the Fe concentration was also measured. In transgenic plants grown at all tested experimental conditions, no changes in Fe concentration relative to the wild-type were noted (Fig. 3). However, in all tested tobacco lines (transgenic and wild-type), the efficiency of Fe translocation to shoots strongly depended on the concentration of elements in the medium. Upon exposure to more diluted medium (1/10 Knop’s), translocation of Fe was more efficient than at 1/2 dilution (Fig. 3c), leading to maintaining Fe at the optimal level in leaves (comparable to those detected in plants grown on 1/2 Knop’s, Fig. 3b), however, at the expense of the Fe level in roots (Fig. 3a). Moreover, the presence of Cd reduced the efficiency of Fe root-to-shoot translocation, but only in plants grown on 1/10 Knop’s medium (Fig. 3c).

#### Expression of tomato metal-homeostasis genes

The expression of *AhHMA4* in tomato altered Zn accumulation and root/shoot distribution in a medium composition-dependent fashion, also in the presence of Cd (Fig. 1). Moreover, in all tested plant lines, similar medium composition-dependent changes in Cd and Fe accumulation were noted (Figs. 2 and 3). Such changes likely result from alteration of the expression pattern of endogenous tomato Zn/Cd/Fe homeostasis genes. Therefore, the expression level of the following genes was examined: (1) *LeIRT1* (Iron-Regulated Transporter 1-like, belonging to the ZIP family) encoding the Fe/Zn/Cd uptake protein (Li et al. 2004); (2) *LeChln (LeNAS)* involved in the regulation of metal cross-homeostasis (including Zn, Cd, and Fe), encoding nicotianamine synthase (NAS) mediating the biosynthesis of nicotianamine (NA), a chelator of several metals (Ling et al. 1999; Takahashi et al. 2003; Curie et al. 2009; Deinlein et al. 2012; Clemens et al. 2013); (3) *LeNRAMP1* (natural resistance-associated macrophage protein, broad substrate range transporters including Fe, Mn, Ni, Cd, Zn, Pb; Nevo & Nelson 2006), the root specific *LeChln*-dependent transporter, for which, in tomato up to now, only its involvement in Fe redistribution from vacuolar stores upon low Fe has been shown (Bereczky et al. 2003); (4) *LeZIP4* (ZRT-IRT-like protein), not yet characterized in tomato, however, from the studies on *Arabidopsis*, maize and rice, it is known that Zn, Fe, and Cd could be involved in *ZIP4* regulation (Ishimaru et al. 2005; 2007; Li et al. 2013; Jain et al. 2013) and that it could be responsible for Zn root/shoot distribution (Ishimaru et al. 2007).

**Fig. 4:**
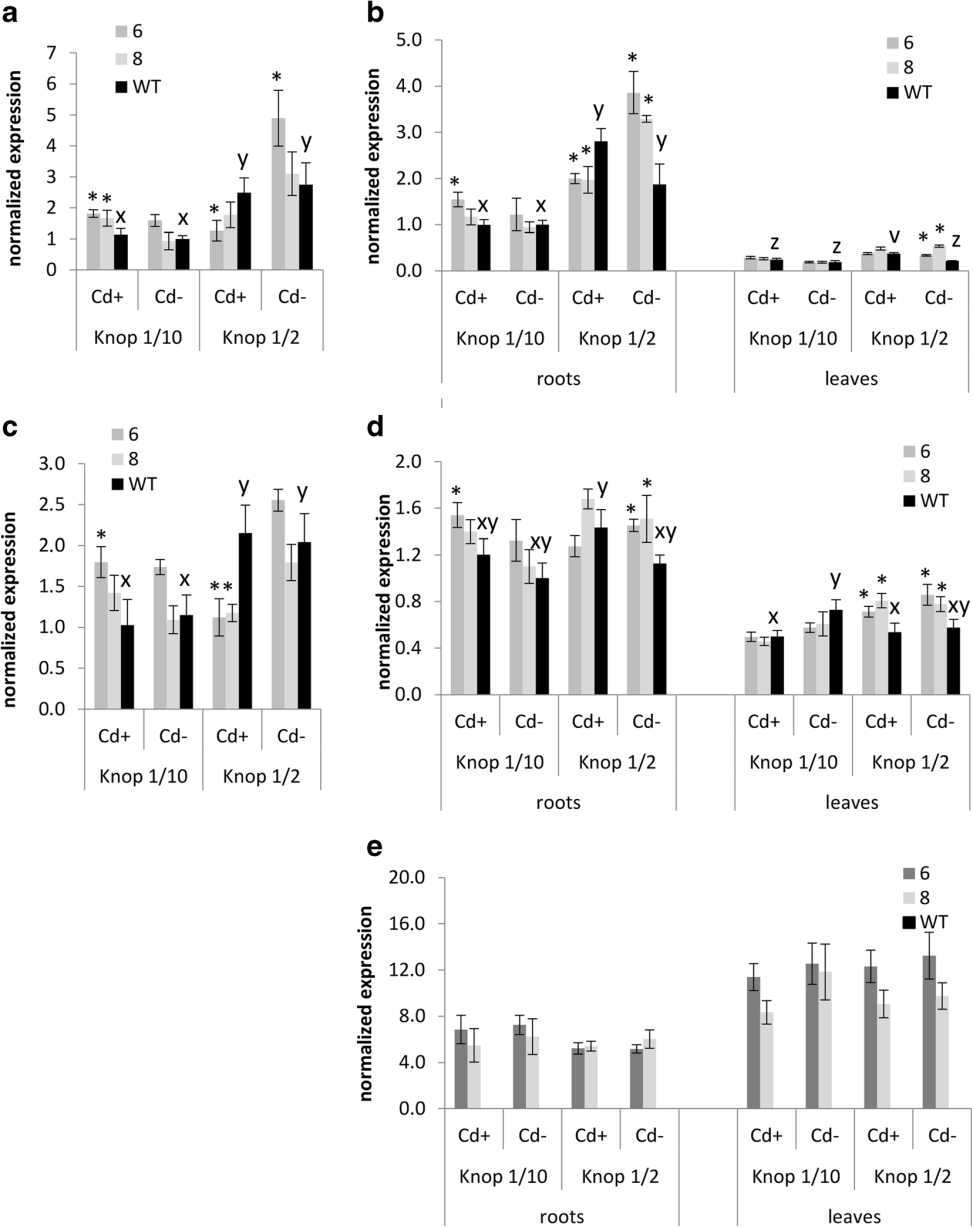
Expression analysis of *LeIRT1* (**a**), *LeChln* (**b**), *LeNRAMP1* (**c**), *LeZIP4* (**d**), and *AhHMA4* (**e**) genes by quantitative real-time RT-PCR (qPCR), in the roots and leaves of 19-day-old *AhHMA4*-transformed tomato plants (*lines 6 and 8*) and wild type (*WT*), grown hydroponically in 1/2 and 1/10 Knop’s medium without Cd or exposed to 0.25 μM Cd for 4 days. Values correspond to arithmetic means ± SD (*n* = 6); values for transgenic plants significantly different from WT at each experimental variant are highlighted by an *asterisk* (*P* ≤ 0.05). *Different letters* represent significantly different values at *P* ≤ 0.05 for wild-type plants grown upon different medium composition; (evaluated by Student’s *t* test)

There were some differences in the expression level between the transgenic and wild-type plants; however, they were rather moderate. Only under certain experimental regimens were the differences greater than twofold (Fig. 4).

In the wild-type plants, the expression level of *LeIRT1*, *LeChln*, and *LeChln*-dependent *LeNramp1* was significantly lower in the roots upon exposure to more diluted medium (1/10 Knop) as compared with 1/2 Knop’s medium, both with and without the presence of Cd (Fig. 4a–c). In the roots of transgenic plants, due to the activity of AhHMA4, the expression of examined tobacco metal-homeostasis genes was modified. The most significant changes were detected in plants grown on 1/2 Knop’s. In the presence of Cd, the level of the mRNA of *LeIRT1*, *LeChln*, and *LeChln*-dependent *LeNramp1* declined compared with the wild-type (Fig. 4a–d). However, on 1/2 Knop’s medium without Cd, the expression of *LeIRT1*, *LeChln*, and also *NtZIP4* was higher than in the wild-type (Fig. 4a, b, d). Much less distinct changes were noted with diluted 1/10 Knop’s medium. In the presence of Cd only, in transgenic’s roots, a moderate increase in the expression of *LeIRT1*, *LeChln*, and *LeNRAMP1* was found. In the leaves, detectable but very low levels of *LeChln* and *LeZIP4* mRNA were noted (Fig. 4b, d).

The expression *AhHMA4* in transgenic tomato was high in both roots and leaves and was not modified by the applied experimental conditions (the degree of medium dilution, presence/absence of Cd). It is worth noting, however, that the expression of this gene was twofold higher in the leaves than in the roots (Fig. 4e).

### Contribution of the expression of *AhHMA4p1::AhHMA4* to the modification of tomato growth, fruit yield, and concentrations of Zn, Cd, and Fe

The aim was to determine how the expression of *AhHMA4* modifies the whole life cycle (vegetative and generative phase) in the presence and absence of Cd, with a view towards biofortification. The mineral composition of the soil and the mineral concentrations in the water extract are shown in Table 2. In the Cd-spiked soil, the total metal concentration was 11.9 mg/kg d.m. (rounded value 12 mg Cd/kg d.m. soil). Growth of plants was terminated after 101 days of cultivation in the soil (in total plants were 123 days old), and selected parameters were evaluated.

**Table 2:** Metals concentration in control and Cd spiked soil used for experiments. Values correspond to means ± SD (*n* = 3)

Metal	Concentration in control soil [mg/kg d.m.]	Concentration in Cd spiked soil [mg/kg d.m.]
Al	799.401 ± 122.313	753.399 ± 81.996
B	2.93768 ± 1.101	3.84114 ± 0.130
Ca	14820.0 ± 1872.04	14650.4 ± 941.511
Cd	0.01524 ± 0.084	11.8917 ± 1.126
Co	0.30995 ± 0.065	0.32262 ± 0.008
Cu	7.09979 ± 1.110	6.55745 ± 0.220
Fe	3897.82 ± 726.31	4556.57 ± 1185.33
K	856.517 ± 97.985	836.531 ± 89.141
Mg	681.128 ± 76.322	694.746 ± 48.018
Mn	39.5671 ± 3.049	34.5474 ± 3.567
Mo	10.794 ± 1.604	9.67959 ± 1.110
Ni	3.75815 ± 0.263	3.31766 ± 0.450
Zn	7.33132 ± 9.507	15.4753 ± 10.185

#### Vegetative phase

The development of plants was monitored throughout the experiment. No differences were detected between transgenic and wild-type plants in their appearance (data not shown). On control soil, the growth rate was comparable between the transgenic and wild-type plants. However, transgenics grew faster on Cd-contaminated soil. Their shoots were significantly higher until the 77th day of growth (Online Resource 2); however, no visual symptoms of Cd toxicity were noted in any line.

#### Generative phase

**Fig. 5:**
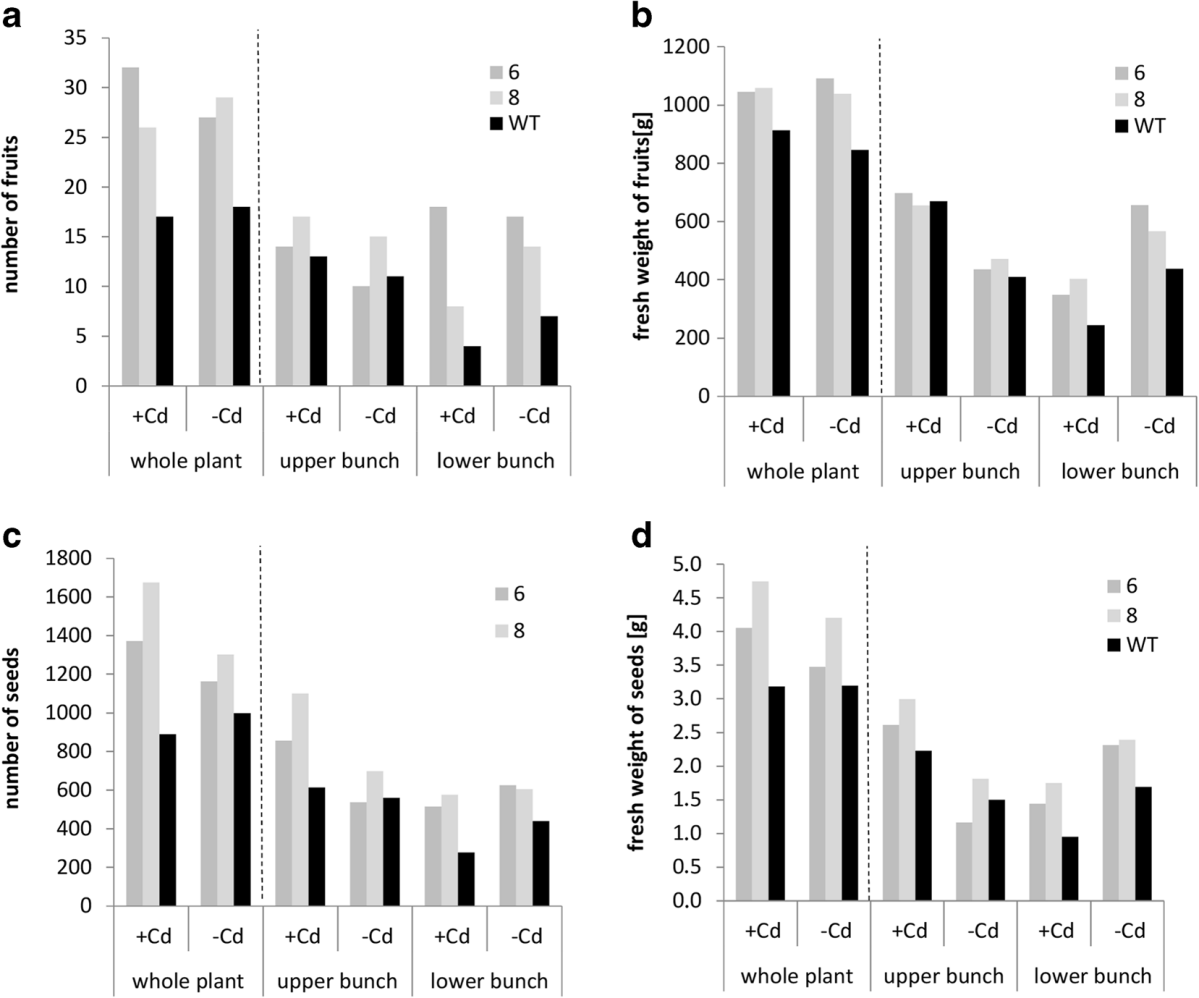
Number of fruits (**a**), fresh weight of fruits (**b**), number of seeds (**c**), and fresh weight of seeds (**d**) collected from tomato plants transformed with *AhHMA4* (*lines 6 and 8*) and from wild type (*WT*) grown for 101 days in soil spiked with 10 mg Cd/kg d.m. and without Cd (control conditions). Each value represents a total number of a given plant organ collected from five plants. Values are given for fruits and seeds collected from the whole plants, upper, and lower bunches

The transgenic plants exhibited a number of features indicating better than wild-type performance in the generative phase, both under the control conditions and in the presence of Cd. The first two, upper and lower, bunches of fruit were analyzed in each plant. The total number of fruits and their biomass were higher in the transgenic than in the wild-type plants (Fig. 5a, b). Similarly, a higher total number and biomass of seeds were noted (Fig. 5c, d). What is noteworthy, the increases in fruit and seed number in transgenics were detected primarily in the lower bunch.

In summary, these data point to the positive impact of the expression of *AhHMA4p1::AhHMA4* on the development (especially in the presence of Cd) of the reproductive organs and, to a lesser extent, on growth in the vegetative phase. This suggests that expression of *AhHMA4* provides a protective effect against Cd when plants are exposed to its low level.

#### Zn, Cd, and Fe concentrations in plant organs

To determine how *AhHMA4p1::AhHMA4* expression in tomato plants modifies Zn, Cd, and Fe accumulation, their concentrations in the roots, leaves, fruits, and seeds were measured.

**Fig. 6:**
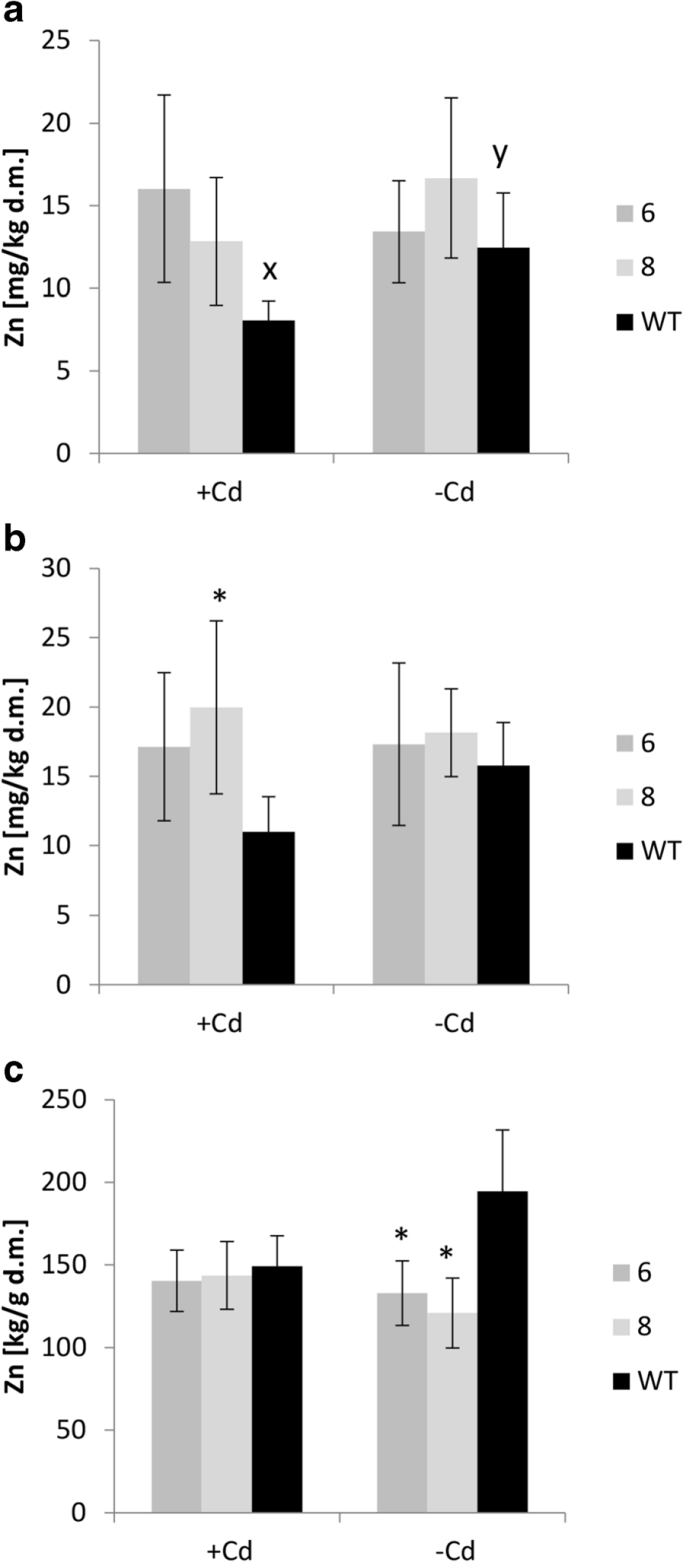
Zn concentration in upper leaves (**a**), lower leaves (**b**), and roots (**c**) of tomato plants expressing *AhHMA4* (*lines 6 and 8*) and wild type (*WT*), grown for 101 days in soil spiked with 10 mg Cd/kg d.m. and without Cd (control soil). Values correspond to arithmetic means ± SD (*n* = 6); values for transgenic plants significantly different from WT at each experimental variant are highlighted by an *asterisk* (*P* ≤ 0.05). *Different letters* represent significantly different values at *P* ≤ 0.05 for wild-type plants grown upon different medium composition (evaluated by Student’s *t* test). Zn concentration from fruits collected from upper and lower bunches, and from seeds collected from these fruits, were not significantly different between transgenic and WT plants. Zn concentrations [mg Zn/kg d.m. of soil] are as follow: fruits from upper bunches: (−Cd) - *Line 6*: 19.05 ± 3.34; *Line 8*: 18.45 ± 0.82; WT: 20.51 ± 2.42, (+ Cd) - *Line 6*: 17.79 ± 9.67; *Line 8*: 18.49 ± 8.04; WT: 18.85 ± 1.32; fruits from lower bunches: (−Cd) - *Line 6*: 18.96 ± 3.30; *Line 8*: 19.16 ± 0.91; WT: 20.46 ± 2.02; (+Cd) - *Line 6*: 15.79 ± 1.27; *Line 8*: 18.18 ± 1.38; WT: 18.42 ± 0.97; seeds from upper bunches: (−Cd) - *Line 6*: 60.71 ± 5.87; *Line 8*: 64.94 ± 5.76; WT: 61.43 ± 6.29; (+Cd) - *Line 6*: 63.48 ± 5.72; *Line 8*: 67.78 ± 2.66; WT: 55.48 ± 3.69, seeds from lower bunches: (−Cd) - *Line 6*: 59.59 ± 7.50; *Line 8*: 61.20 ± 8.25; WT: 58.12 ± 5.03; (+Cd) - *Line 6*: 61.39 ± 7.25; *Line 8*: 61.98 ± 3.67; WT: 60.65 ± 7.24

**Fig. 7:**
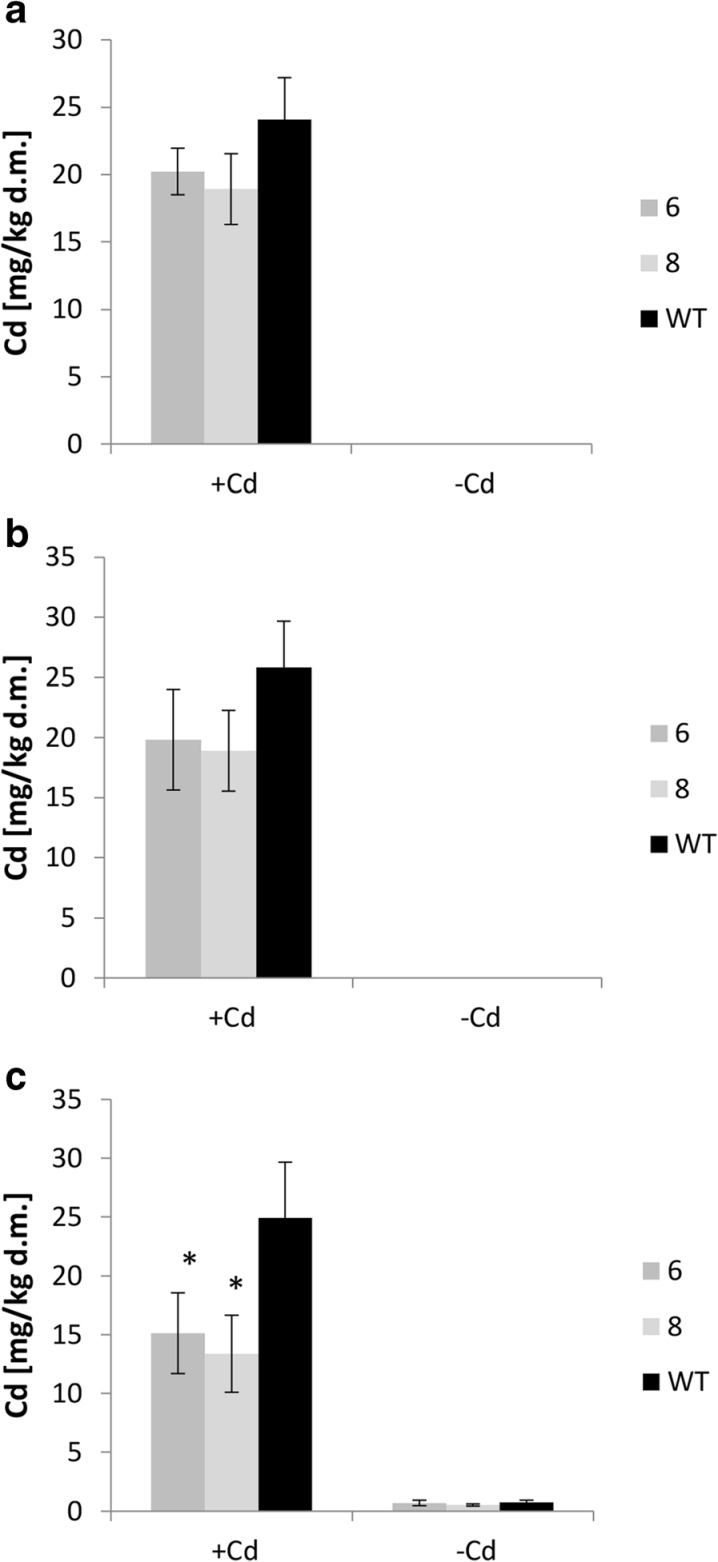
Cd concentration in upper leaves (**a**), lower leaves (**b**), roots (**c**) of tomato plants expressing *AhHMA4* (*lines 6 and 8*) and wild type (*WT*), grown for 101 days in soil spiked with 10 mg Cd/kg d.m. and without Cd (control medium). Values correspond to means ± SD (*n* = 3); values for transgenic plants significantly different from the WT at each experimental variant are indicated by an *asterisk* (*P* ≤ 0.05) (evaluated by Student’s *t* test). Cd concentration from fruits collected from upper and lower bunches, and from seeds collected from these fruits, were not significantly different between transgenic and WT plants. Cd concentrations [mg Cd/kg d.m. of soil] are as follow: fruits form upper bunches - *Line 6*: 0.14 ± 0.07; *Line 8* 0.24 ± 0.08; WT: 0.23 ± 0.05, fruits from lower bunches: − *Line 6*: 0.11 ± 0.10; *Line 8*: 0.20 ± 0.07; WT: 0.32 ± 0.12, seeds form upper bunches: − *Line 6*: 0.77 ± 0.15; *Line 8*: 1.30 ± 0.54; WT: 1.01 ± 0.15, seeds from lower bunches: − *Line 6*: 0.73 ± 0.076; *Line 8*: 1.21 ± 0.55; WT: 0.86 ± 0.19

Expression of *AhHMA4* contributed to moderately enhanced Zn level in the leaves, but only in the presence of Cd (Fig. 6a, b). No changes were detected in the fruits or seeds, except for a small increase in the seeds developed in fruits from the 2nd bunch (data in the legend to Fig. 6). In the roots, the opposite effect was noted, i.e., reduction of the Zn concentration in the transgenic plants grown on control soil (Fig. 6c). Importantly, the Cd concentration was reduced in almost all examined organs of transgenic plants. It was lower in the upper and lower leaves (Fig. 7a, b), roots (Fig. 7c), and also in fruits collected from the upper bunches (data in the legend to Fig. 7). The Fe concentration was not significantly modified in the transgenic plants. Moreover, there were no differences between treatments (plants from control soil and from Cd-spiked soil; Online Resource 3).

## Discussion

The HMA4 transporter from *A. thaliana* or *A. halleri* is not fully specific for Zn, as it mediates Cd transport as well (Verret et al. 2004; Hanikenne et al. 2008). Its involvement in the control of Zn root-to-shoot translocation, but also toxic Cd, was considered a serious shortcoming for its use in strategies of genetic biofortification in minerals. In contrast, this quality was seen as an advantage for the use of *HMA4* in phytoremediation to engineer a plant with high level of both metals in aerial parts. However, contrary to expectations, expression of *AhHMA4p1::AhHMA4* or 35S::*AtHMA4* in tobacco reduced Cd concentrations in both roots and shoots (Barabasz et al. 2010; Siemianowski et al. 2011). It is known that the rate of root-to-shoot translocation of metals is a prerequisite, determining not only the level of a metal’s accumulation in shoots, but also in the generative organs (Palmgren et al. 2008; Waters & Sankaran 2011). Therefore, it was assumed that expression of *AhHMA4p1::AhHMA4* from *A. halleri* in tomato could lead to more efficient translocation of Zn to aerial plant parts, likely without the risk of enhancing the Cd content upon growth in a contaminated environment. Indeed, in transgenic tomato, the Zn concentration was found to be significantly higher in the leaves and stems (Barabasz et al. 2012). However, increase in the Zn concentration in the aerial parts of transgenic plants was noted only upon exposure to 10 μM Zn, and not at other Zn levels (1 and 20 μM). Thus, the expression of *AhHMA4* contributed to enhancement of Zn translocation, but not at all tested Zn concentrations in the medium. Metal supply dependence of metal root/shoot distribution, different in the transgenic and wild types, has also been shown in plants expressing 35S:*AtHMA4*, as well as other metal transporters such as *AtCAXs*, *AtECA3*, and *HvHMA2* (Korenkov et al. 2007; Barabasz et al. 2011; 2013).

Here, we have shown that the pattern of the modification of Zn root/shoot distribution due to *AhHMA4p1::AhHMA4* expression also depended on the degree of dilution of all medium components. In transgenic plants, Zn translocation to shoots was more efficient than in the wild type, and the Zn concentration in leaves was higher when plants were cultivated on highly diluted 1/10 Knop’s medium, especially in the presence of Cd (which was not the case with 1/2 Knop) (Fig. 1). These changes were accompanied by enhanced expression of *LeIRT1* (Fig. 4). Higher expression of *LeIRT1* in transgenic tomato grown on diluted 1/10 Knop (containing also low Fe concentraion) might suggest that Fe deficiency-inducible *IRT1*, as a broad substrate range transporter mediating distribution of not only Fe, but also of Zn, Mn, Cd, and Ni, primarily at low Fe (Eckhardt et al. 2001) could contribute to a certain extent to the detected enhancement of Zn accumulation in transgenic plants. Moreover, it was shown that, at 1/10 Knop, the expression of *LeChln* (*LeNAS* involved in synthesis of nicotianamine, NA) and *LeNRAMP1* was also higher than in wild-type plants (Fig. 4a–c), which is consistent with the regulation of *LeIRT1* and *LeNRAMP1* being dependent on *LeChln* (Stephan & Scholz 1993). *LeChln* is the only *NAS* gene in tomato involved in biosynthesis of nicotianamine (NA), chelator of metals (including Zn, Fe, and probably Cd). Recent study implicated a role of *AhNAS2* in Zn and Cd translocation to shoots of *A. halleri*. A decrease in NA concentration in roots through RNAi targeting *NAS2* resulted in reduction in Zn and Cd level in leaves (Deinlein et al. 2012). In tomato, the role of *LeChln* was not studied extensively up to date. It was shown that adequate Fe uptake and distribution in tomato plants depended on *LeChln*; however, it is not regulated by Fe level (Ling et al. 1999; Bereczky et al. 2003). Moreover, in S-deficient tomato, its expression was abolished (Zuchi et al. 2009). Here, it was shown that *LeChln* is regulated by the level of dilution of the medium. Its expression was lower in roots of plants grown at highly diluted medium (1/10 Knop’s medium) compared with 1/2 Knop’s medium, and the presence of Cd did not affect the transcript level (Fig. 4b). The third gene, *LeNRAMP1*, belongs to a group of membrane importer proteins which exhibit functional divergence and broad substrate specificity including Fe, Mn, Ni, Cd, Zn, and Pb (Nevo and Nelson 2006). However, there are numerous uncertainties about the physiological role of *LeNRAMP1* in tomato and its regulation. Experiments with a range of Fe concentrations in the medium demonstrated that it is expressed primarily in tomato roots at higher level upon Fe deficiency conditions. Yeast-based experiments show its presence in intracellular vesicles, tonoplast, and partially in plasma membrane. The tomato *NRAMP* genes were not tested for their substrate specificity with the use of a range of yeast mutants. Complementation analysis showed only that *LeNRAMP1* encodes a metal transporter able to restore growth of a yeast metal uptake mutant *smf1* (Bereczky et al. 2003). To compare, it was shown that *NRAMP1* from *Malus baccata* transports Fe, Mn, and Cd (Xiao et al. 2008) and from *A. thaliana* Fe, Zn, and Co (Cailliatte et al. 2010). Thus, detailed analysis to fully characterize *LeNRAMP1* is necessary to understand its physiological function in tomato and contribution to the modification of Zn/Cd root/shoot accumulation and distribution in *HMA4*-expressing plants. Here, it was shown that ectopic expression of *AhHMA4* in tomato leads to coordinated response of its endogenous genes involved in metals uptake (*LeIRT1*), in uptake and/or redistribution (*LeNRAMP1, LeZIP4*), and in the regulation of metal cross-homeostasis (*LeChln*), likely as a response to alteration of a plant metal status due to export activity of HMA4 (Barabasz et al. 2012; Siemianowski et al. 2013). It is becoming evident that modifications of an endogenous metal homeostasis network due to transgene expression constitute a key factor in the development of characteristic features of transformants (Antosiewicz et al. 2014). In the *HMA4*-expressing plants, the apoplast/symplast Zn status changed due to the export activity of the HMA4 protein, resulting in ion imbalances at the cellular/tissue level, which likely induced endogenous metal homeostasis mechanisms to regain balance (Siemianowski et al. 2014; Barabasz et al. 2012; Kendziorek et al. 2014). Since the molecular characteristics of the host plant are specific for given growing conditions (a metal status, overall medium composition), the transgene activity takes place against different molecular backgrounds. It has been proposed that this interplay leads to the medium-dependent metal-related phenotype of transgenic plants.

Modification of the expression pattern of *LeIRT1, LeChln*, and *LeNRAMP1* in transgenic tomato was also noted in plants cultivated at 1/2 diluted medium. In the roots of transgenics grown in the presence of 0.25 μM Cd, their expression was significantly lower than in the wild type. On the other hand, higher expression of *LeChln* than in the wild type was detected in the roots of transgenic plants grown without Cd (Fig. 4b). Under these conditions, however, only minor modification of Zn accumulation and root/shoot distribution in transgenic plants was detected, and accumulation of Cd and Fe was not different from the wild type (Figs. 2 and 3). Therefore, the differing expression pattern of *LeChln* in transgenic and wild-type plants grown at 1/2 Knop probably reflects changes in the status of Zn, Fe, and probably other metals due to the export activity of AhHMA4, as cross-homeostasis mechanisms have been shown to be affected in *HMA4*-expressing plants (Antosiewicz et al. 2014). The molecular mechanisms of the regulation of metal homeostasis are the best characterized in *A. thaliana*; however, in tomato, these processes are poorly understood as only some tomato genes involved in metal transport have been cloned and characterized.

Interestingly, in transgenic tomato expressing *AhHMA4* under its own native promoter (*AhHMA4*_*p1*_), the level of expression of the transgene was lower in roots than in leaves (Fig. 4e). In the previous study based on less sensitive RT-PCR, transcript level was also higher in leaves compared to roots, although expression was compared in plants grown in different media—in 1/4 Knop’s medium supplemented with 1 and 10 μM Zn (Barabasz et al. 2012). Results indicate that in transgenic tomato, expression of *AhHMA4p1:: AhHMA4* is not regulated by Zn, as in *A. halleri*.

In *A. halleri*, however, expression of *AhHMA4* is higher in the roots compared with shoots (Talke et al. 2006). Thus, in transgenic tomato, the regulation of the *A. halleri* promoter is different than in the native plant. In tomato, the *HMA4* gene has not yet been cloned.

The expression of *AhHMA4* did not alter the Cd accumulation pattern under the medium conditions used in our experiments. However, all tested lines (transgenic and wild-type) accumulated more Cd when grown on the more diluted medium (1/10) (Fig. 2). This is in agreement with the reports on more effective entry of Cd into plants from poor soils compared with rich ones and from more diluted liquid media. It is known that plants do not have transporters specific for Cd. This toxic metal crosses plant membranes through pathways for other minerals, including Fe, Zn, and Ca. Competition between metals for binding sites of metal transporters results in a protective effect of the presence of these metal cations against contamination of plants with toxic Cd, as their higher levels inhibit Cd uptake (Kudo et al. 2007; Ueno et al. 2008; DalCorso et al. 2008; Gallego et al. 2012; Tan et al. 2013).

### Expression of *AhHMA4p1::AhHMA4* in tomato contributes to better performance of soil-grown plants in the presence and absence of Cd

The advantage of using liquid media for plant growth is the ability to control the mineral composition, which is necessary to learn about specific plant responses at the transcriptome and metabolome levels to the conditions that have been set up. However, it is known that plants grown in nature have to cope with complex, variable soil conditions. These include interactions with microorganisms and the composition of the soil solution, which depends on the content of minerals, presence of organic matter, as well as water availability. For example, the reduction of soil salinity decreased distribution of heavy metals by tomato and amaranth (Li et al. 2012). It has also been demonstrated that an increased mineral residual phase in relation to the organic-bound phase may reduce the bioavailability of Cr, Ni, Cu, As, Cd, and Pb for crops such as tomato or pumpkin (Islam et al. 2014). The above confirms that soil conditions generate an environment for plant growth that is very different from that of hydroponic conditions. The results obtained in the course of hydroponic experiments do not, therefore, necessarily reflect the same processes occurring in soil. For example, basic qualitative and quantitative differences were demonstrated in arsenic uptake, root-to-shoot translocation, accumulation pattern, and detoxification mechanisms between plants grown under soil and hydroponic conditions (Zabłudowska et al. 2009).

Here, we have shown that expression of *AhHMA4p1::AhHMA4* in tomato improved plant productivity, both in control and Cd-contaminated soil. The total number and biomass of fruits and seeds were higher in transgenic than in wild-type plants. Importantly, the detected differences resulted primarily from increased fruit yield within the first (lower) bunch of transgenic plants and, not the second, upper one (Fig. 5). These changes were accompanied by slightly enhanced (though not statistically significantly) Zn concentrations in the leaves (Fig. 6a, b). Most importantly, the Cd concentration in the leaves and roots of transgenic plants was significantly lower compared with the wild type (Fig. 7a–c), which is in agreement with previous reports coming from hydroponic experiments on the decrease in the Cd content in the roots and leaves due to the expression of *HMA4* (Barabasz et al. 2010; Siemianowski et al. 2011). However, expression of *AhHMA4* did not significantly affect the Cd concentration in the fruits. Moreover, the Zn and Fe concentrations in the fruits and seeds remained unaltered relative to the wild type (Figs. 6–7, Online Resource 3). Thus, the increase in the fruit yield detected in the transgenic plants under control conditions and in the presence of Cd likely resulted from the better supply of Zn to leaves and from the restriction of Cd flux into the aerial vegetative plant parts. Such modifications are important for the tomato. It has been shown that exposure to Cd, even in low to moderate doses, decreases the growth of tomato plants and fruit yield (Gratão et al. 2008). Although Cd is known to accumulate mainly in roots (Hediji et al. 2010; Gallego et al. 2012), certain tomato cultivars have the ability to translocate large amounts of Cd to aerial plant parts, including fruits. For example, in tomato cv. MicroTom, in plants exposed to Cd, the highest Cd concentrations were detected in fruits. Upon long-term Cd treatment, the Cd concentration reached up to 2–5 mmol Cd g^−1^ d.w. (Gratão et al. 2008). This generates a threat of this toxic element entering the food chain, since the tomato, as a healthy food containing high amounts of nutrients and secondary metabolites, is an important crop food. Cd is still present in agricultural soils; therefore, elimination of a threat of Cd accumulation in fruits upon long-term exposure even to its low doses is of great importance (Arruda & Azevedo 2009).

In summary, expression of *AhHMA4* under its own promoter in tomato resulted in more efficient translocation of Zn and in restriction of Cd translocation to shoots. Nevertheless, the processes leading to loading of metals into fruits were not significantly stimulated; as a result, the produced fruits, and also seeds, were not fortified with Zn. However, improved fruit yield and performance of transgenic plants in the vegetative stage, and higher Zn concentration in the leaves (when cultivated on control and Cd-contaminated soil), suggest the usefulness of this transformation for improving the growth of tomato on low-Zn soil, also contaminated with Cd, with a chance of reducing the concentration of this toxic metal in the aerial parts.

## Electronic supplementary material

Below is the link to the electronic supplementary material.

ESM 1 Dry biomass of roots (**a**) and leaves (**b**) of 19-day-old tomato plants expressing *AhHMA4* (*lines 6, 8*), and wild-type (*WT*) grown hydroponically in 1/2 and 1/10 Knop’s medium without and with 0.25 μM Cd for 4 days. Values correspond to arithmetic means + SD (*n* = 6). (PDF 51.5 kb)

ESM 2 Height of tomato plants expressing *AhHMA4* (*lines 6, 8*), and wild-type (*WT*), grown fopr 101 days in control soil (**a**), spiked with 10 mg Cd/kg d.m. (**b**). Values correspond to means ± SD (n = 3); those significantly different from the WT (Student’s *t* test) are indicated by *arrows* (*P* ≤ 0.05). (PDF 60.7 kb)

ESM 3 Fe concentration in upper leaves (**a**), lower leaves (**b**), roots (**c**) of tomato plants expressing *AhHMA4* (*lines 6, 8*), and wild-type (*WT*), grown for 101 days in soil spiked with 10 mg Cd/kg d.m. and without Cd (control soil). Values correspond to means ± SD (*n* = 3); *Different letters* represent significantly different values at P < 0.05 for wild-type plants grown upon different medium composition; (evaluated by Student’s *t* test). Fe concentration from fruits collected from upper and lower bunches, and from seeds collected from these fruits, were not significantly different between transgenic and WT plants. Fe concentrations [mg/kg d.m..] are as follow: fruits form upper bunches: (-Cd) *Line 6:* 50,89 ± 9,54; *Line 8:* 42,22 ± 3,17; WT 55,85 ± 15,69; (+Cd) *Line 6:* 47,78 ± 9,67; *Line 8:* 49,85 ± 8,04; WT 50,30 ± 4,13;fruits from lower bunches: (-Cd) *Line 6:* 44,84 ± 7,02; *Line 8:* 38,83 ± 3,51; WT 49,60 ± 7,16; (+Cd) *Line 6:* 42,76 ± 12,64; *Line 8:* 40,67 ± 5,25; WT 49,57 ± 0,42, seeds form upper bunches: ( -Cd) *Line 6:* 76,03 ± 37,96; *Line 8:* 68,93 ± 7,27; WT 72,67 ± 24,22; (+Cd) *Line 6:* 40,21 ± 4,47; *Line 8:* 88,84 ± 14,97; WT 37,55 ± 4,92, seeds from lower bunches: (-Cd) *Line 6:* 48,14 ± 4,48; *Line 8:* 67,34 ± 18,49; WT 41,65 ± 6,86, (+Cd) *Line 6:* 59,65 ± 9,43; *Line 8:* 52,95 ± 3,67; WT 42,76 ± 14,06. (PDF 53.1 kb)
