# Supplementary material for: Determination the Usefulness of AhHMA4p1::AhHMA4 Expression in Biofortification Strategies
Source: Water Air Soil Pollut. 2016 May 23;227:186. doi: 10.1007/s11270-016-2877-0 (PMC4877419; doi:10.1007/s11270-016-2877-0)
Supplement: Supplementary file 1 — Dry biomass of roots (a) and leaves (b) of 19-day-old tomato plants expressing AhHMA4 (lines 6, 8), and wild-type (WT) grown hydroponically in 1/2 and 1/10 Knop’s medium without and with 0.25 μM Cd for 4 days. Values correspond to arithmetic means + SD (n = 6). (PDF 51.5 kb) [file 11270_2016_2877_MOESM1_ESM.pdf]

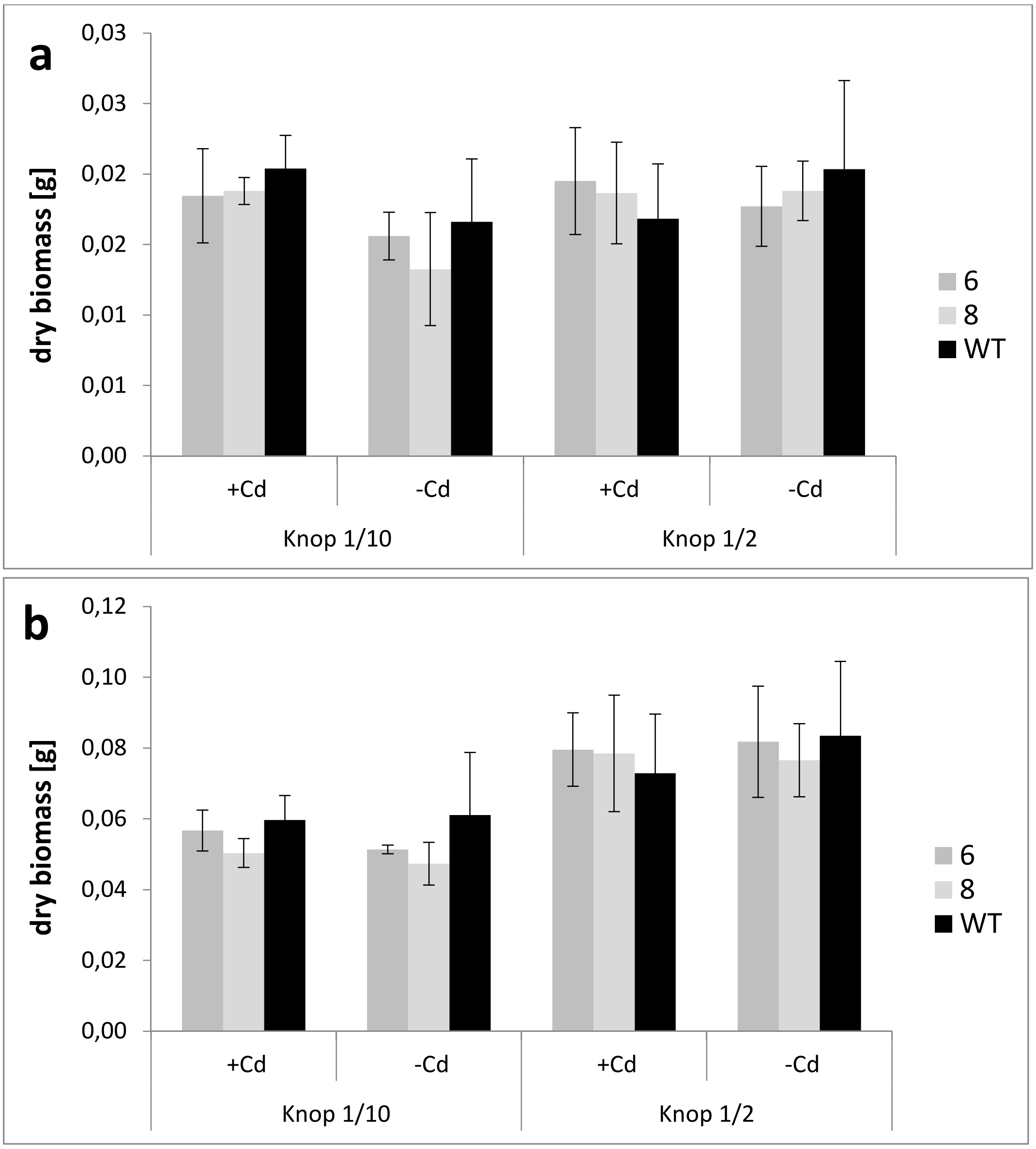

Online Resource 1. Dry biomass of roots (a) and leaves (b) of 19-day-old tomato plants expressing *AhHMA4* (lines 6, 8), and wild-type (WT) grown hydroponically in 1/2 and 1/10 Knop's medium without and with 0.25 µM Cd for 4 days. Values correspond to arithmetic means ± SD (*n* = 6);

Determination the usefulness of *AhHMA4p1::AhHMA4* expression in biofortification strategies

Water, Air and Soil Pollution

Aleksandra Weremczuk[1], Anna Barabasz[1], Anna Ruszczyńska[2], Ewa Bulska[2] and Danuta Maria Antosiewicz[1*]

[1]University of Warsaw, Faculty of Biology, Warszawa, Poland.

[2]University of Warsaw, Faculty of Chemistry, Warszawa, Poland.

[*] corresponding author: Danuta Maria Antosiewicz, email: dma@biol.uw.edu.pl
