# Supplementary material for: Determination the Usefulness of AhHMA4p1::AhHMA4 Expression in Biofortification Strategies
Source: Water Air Soil Pollut. 2016 May 23;227:186. doi: 10.1007/s11270-016-2877-0 (PMC4877419; doi:10.1007/s11270-016-2877-0)
Supplement: Supplementary file 2 — Height of tomato plants expressing AhHMA4 (lines 6, 8), and wild-type (WT), grown fopr 101 days in control soil (a), spiked with 10 mg Cd/kg d.m. (b). Values correspond to means ± SD (n = 3); those significantly different from the WT (Student’s t test) are indicated by arrows (P ≤ 0.05). (PDF 60.7 kb) [file 11270_2016_2877_MOESM2_ESM.pdf]

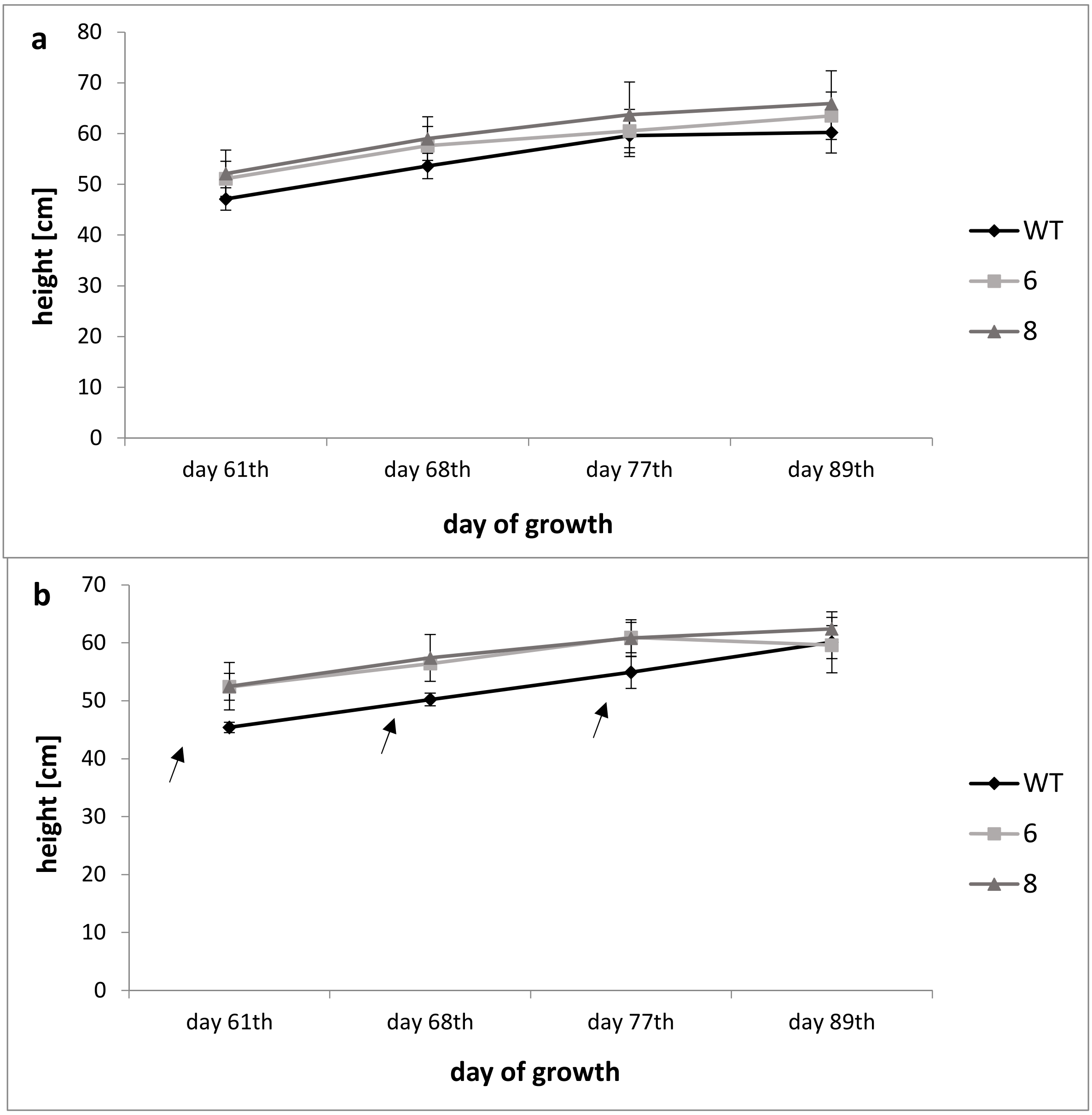

Online Resource 2. Height of tomato plants expressing *AhHMA4* (lines 6, 8), and wild-type (WT), grown for 101 days in control soil (a), and spiked with 10 mg Cd/kg d.m. (b). Values correspond to means ± SD (n=3); those significantly different from the WT (Student's *t*-test) are indicated by arrows ($P \leq 0.05$).

Determination the usefulness of *AhHMA4p1::AhHMA4* expression in biofortification strategies.

Water, Air and Soil Pollution

Aleksandra Weremczuk[1], Anna Barabasz[1], Anna Ruszczyńska[2], Ewa Bulska[2] and Danuta Maria Antosiewicz[1*]

[1]*University of Warsaw, Faculty of Biology, Warszawa, Poland.*

[2]*University of Warsaw, Faculty of Chemistry, Warszawa, Poland.*
