# Supplementary material for: Determination the Usefulness of AhHMA4p1::AhHMA4 Expression in Biofortification Strategies
Source: Water Air Soil Pollut. 2016 May 23;227:186. doi: 10.1007/s11270-016-2877-0 (PMC4877419; doi:10.1007/s11270-016-2877-0)
Supplement: Supplementary file 3 — Fe concentration in upper leaves (a), lower leaves (b), roots (c) of tomato plants expressing AhHMA4 (lines 6, 8), and wild-type (WT), grown for 101 days in soil spiked with 10 mg Cd/kg d.m. and without Cd (control soil). Values correspond to means ± SD (n = 3); Different letters represent significantly different values at P < 0.05 for wild-type plants grown upon different medium composition; (evaluated by Student’s t test). Fe concentration from fruits collected from upper and lower bunches, and from seeds collected from these fruits, were not significantly different between transgenic and WT plants. Fe concentrations [mg/kg d.m..] are as follow: fruits form upper bunches: (-Cd) Line 6: 50,89 ± 9,54; Line 8: 42,22 ± 3,17; WT 55,85 ± 15,69; (+Cd) Line 6: 47,78 ± 9,67; Line 8: 49,85 ± 8,04; WT 50,30 ± 4,13;fruits from lower bunches: (-Cd) Line 6: 44,84 ± 7,02; Line 8: 38,83 ± 3,51; WT 49,60 ± 7,16; (+Cd) Line 6: 42,76 ± 12,64; Line 8: 40,67 ± 5,25; WT 49,57 ± 0,42, seeds form upper bunches: ( -Cd) Line 6: 76,03 ± 37,96; Line 8: 68,93 ± 7,27; WT 72,67 ± 24,22; (+Cd) Line 6: 40,21 ± 4,47; Line 8: 88,84 ± 14,97; WT 37,55 ± 4,92, seeds from lower bunches: (-Cd) Line 6: 48,14 ± 4,48; Line 8: 67,34 ± 18,49; WT 41,65 ± 6,86, (+Cd) Line 6: 59,65 ± 9,43; Line 8: 52,95 ± 3,67; WT 42,76 ± 14,06. (PDF 53.1 kb) [file 11270_2016_2877_MOESM3_ESM.pdf]

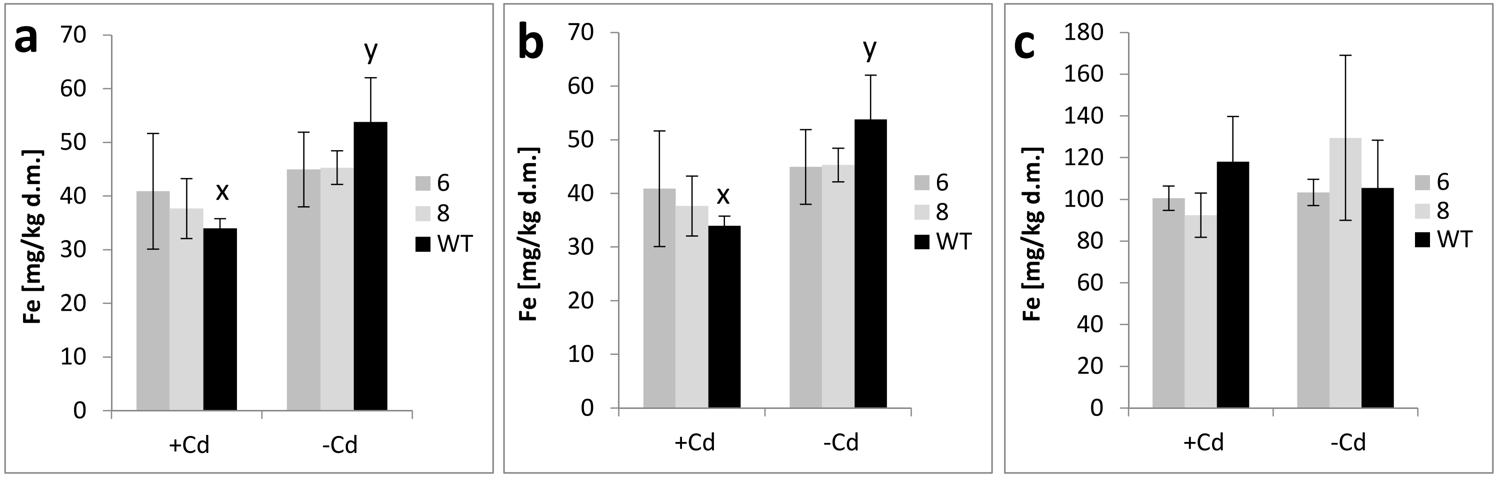

Online Resource 3. Fe concentration in upper leaves (a), lower leaves (b), roots (c) of tomato plants expressing *AhHMA4* (lines 6, 8), and wild-type (WT), grown for 101 days in soil spiked with 10 mg Cd/kg d.m. and without Cd (control soil). Values correspond to means ± SD (n=3); Different letters represent significantly different values at $P < 0.05$ for wild-type plants grown upon different medium composition; (evaluated by Student's *t*-test).

Fe concentration from fruits collected from upper and lower bunches, and from seeds collected from these fruits, were not significantly different between transgenic and WT plants. Fe concentrations [mg/kg d.m..] are as follow:

fruits form upper bunches: (-Cd) Line 6: 50,89±9,54; Line 8: 42,22±3,17; WT 55,85±15,69; (+Cd) Line 6: 47,78±9,67; Line 8: 49,85±8,04; WT 50,30±4,13;
fruits from lower bunches: (-Cd) Line 6: 44,84±7,02; Line 8: 38,83±3,51; WT 49,60±7,16; (+Cd) Line 6: 42,76±12,64; Line 8: 40,67±5,25; WT 49,57±0,42 ,
seeds form upper bunches: (-Cd) Line 6: 76,03±37,96; Line 8: 68,93±7,27; WT 72,67±24,22; (+Cd) Line 6: 40,21±4,47; Line 8: 88,84±14,97; WT 37,55±4,92 ,
seeds from lower bunches: (-Cd) Line 6: 48,14±4,48; Line 8: 67,34±18,49; WT 41,65±6,86, (+Cd) Line 6: 59,65±9,43; Line 8: 52,95±3,67; WT 42,76±14,06.

Determination the usefulness of *AhHMA4p1::AhHMA4* expression in biofortification strategies.
Water, Air and Soil Pollution

Aleksandra Weremczuk[1], Anna Barabasz[1], Anna Ruszczyńska[2], Ewa Bulska[2] and Danuta Maria Antosiewicz[1*]
[1]*University of Warsaw, Faculty of Biology, Warszawa, Poland.*
[2]*University of Warsaw, Faculty of Chemistry, Warszawa, Poland.*
[*] *corresponding author: Danuta Maria Antosiewicz, email: dma@biol.uw.edu.pl*
